# Prevalence and Risk Factors for Schistosomiasis among Schoolchildren in two Settings of Côte d’Ivoire

**DOI:** 10.3390/tropicalmed4030110

**Published:** 2019-07-23

**Authors:** Etienne K. Angora, Jérôme Boissier, Hervé Menan, Olivier Rey, Karim Tuo, Andre O. Touré, Jean T. Coulibaly, Aboulaye Méité, Giovanna Raso, Eliézer K. N’Goran, Jürg Utzinger, Oliver Balmer

**Affiliations:** 1 Swiss Tropical and Public Health Institute, P.O. Box, CH-4002 Basel, Switzerland; 2 University of Basel, P.O. Box, CH-4003 Basel, Switzerland; 3 Unité de Formation et de Recherche Sciences Pharmaceutiques et Biologiques, Université Félix Houphouët-Boigny, Abidjan BPV 34, Côte d’Ivoire; 4 IHPE, Univ. Montpellier, CNRS, Ifremer, Univ. Perpignan Via Domitia, 66860 Perpignan, France; 5 Institut Pasteur de Côte d’Ivoire, Abidjan BPV 490, Côte d’Ivoire; 6 Centre Suisse de Recherches Scientifiques en Côte d’Ivoire, 01 BP 1303, Abidjan 01, Côte d’Ivoire; 7 Unité de Formation et de Recherche Biosciences, Université Félix Houphouët-Boigny, 22 BP 770, Abidjan 22, Côte d’Ivoire; 8 Programme National de Lutte contre les Maladies Tropicales Négligées à Chimiothérapie Préventive, 06 BP 6394, Abidjan 06, Côte d’Ivoire

**Keywords:** Côte d’Ivoire, prevalence, risk factors, *Schistosoma haematobium*, *Schistosoma mansoni*, schistosomiasis

## Abstract

Schistosomiasis is a parasitic disease affecting more than 250 million people, primarily in sub-Saharan Africa. In Côte d’Ivoire both *Schistosoma haematobium* (causing urogenital schistosomiasis) and *Schistosoma mansoni* (causing intestinal schistosomiasis) co-exist. This study aimed to determine the prevalence of *S. haematobium* and *S. mansoni* and to identify risk factors among schoolchildren in the western and southern parts of Côte d’Ivoire. From January to April 2018, a cross-sectional study was carried out including 1187 schoolchildren aged 5–14 years. Urine samples were examined by a filtration method to identify and count *S. haematobium* eggs, while stool samples were subjected to duplicate Kato-Katz thick smears to quantify eggs of *S.* *mansoni* and soil-transmitted helminths. Data on sociodemographic, socioeconomic, and environmental factors were obtained using a pretested questionnaire. Multivariate logistic regression was employed to test for associations between variables. We found a prevalence of *S. haematobium* of 14.0% (166 of 1187 schoolchildren infected) and a prevalence of *S. mansoni* of 6.1% (66 of 1089 schoolchildren infected). In the southern part of Côte d’Ivoire, the prevalence of *S. haematobium* was 16.1% with a particularly high prevalence observed in Sikensi (35.6%), while *S. mansoni* was most prevalent in Agboville (11.2%). Swimming in open freshwater bodies was the main risk factor for *S. haematobium* infection (adjusted odds ratio (AOR) = 127.0, 95% confidence interval (CI): 25.0–634.0, *p* < 0.001). Fishing and washing clothes in open freshwater bodies were positively associated with *S. haematobium* and *S. mansoni* infection, respectively. Preventive chemotherapy using praziquantel should be combined with setting-specific information, education, and communication strategies in order to change children’s behavior, thus avoiding contact with unprotected open freshwater.

## 1. Introduction

Schistosomiasis is a water-based chronic parasitic disease caused by trematode worms of the genus *Schistosoma*. Considered as a neglected tropical disease by the World Health Organization (WHO), schistosomiasis affects more than 250 million people worldwide with an estimated global burden of 1.4 million disability-adjusted life years (DALYs) in 2017 [1,2,3]. Schistosomiasis remains a public health problem in countries of the tropics and subtropics with approximately 90% of cases concentrated in Africa [3,4]. Humans are the definitive host for adult parasites, while specific freshwater snails act as intermediate hosts [3,4]. Hence, the transmission of schistosomiasis is governed by social-ecological systems (e.g., conditions of poverty and living near open freshwater bodies) [5]. Schistosome eggs are excreted by humans with feces or urine. After hatching, miracidia infect specific snails to produce cercariae. Schistosome cercariae penetrate the unbroken skin of humans during domestic (e.g., washing clothes or dishes) and recreational activities (e.g., bathing and swimming in unprotected open freshwater bodies). Various factors have been shown to facilitate transmission of schistosomiasis in Africa, such as living in close proximity to freshwater bodies (e.g., rivers, small dams, irrigation schemes, and lakes), socioeconomic factors which influence occupational activities (e.g., poor people without running water at home are likely to contact freshwater bodies) and climate change [6,7,8]. The lack of access to improved sanitation contributes to open defecation, which results in environmental contamination that enhances the transmission of schistosomiasis [9].

In Côte d’Ivoire, snails of the genera *Biomphalaria* and *Bulinus* are the intermediate hosts for *Schistosoma mansoni* and *Schistosoma haematobium*, respectively [10]. While both *S. mansoni* and *S. haematobium* are endemic in Côte d’Ivoire [11], the former species is predominantly found in the western part of the country [12,13] and *S. haematobium* is mostly present in the central and southern parts [9,14]. In northern Côte d’Ivoire, a recent study reported low prevalence rates of 1.9% and 3.5% for *S. haematobium* and *S. mansoni* among school-aged children, respectively [15]. To enhance control efforts and shift the focus from morbidity control toward interruption of transmission, fine-grained information on the distribution of the disease is important, including underlying risk factors.

The purpose of this study was to determine the prevalence of schistosomiasis and risk factors among schoolchildren in the western and southern parts of Côte d’Ivoire where the disease is most prevalent. The results will assist public health authorities of Côte d’Ivoire to refine control measures and complement preventive chemotherapy with specific information about infection prevalence and intensity, and to enhance education and communication approaches that are readily tailored to specific social-ecological contexts.

## 2. Materials and Methods

### 2.1. Study Settings and Population

**Figure 1:**
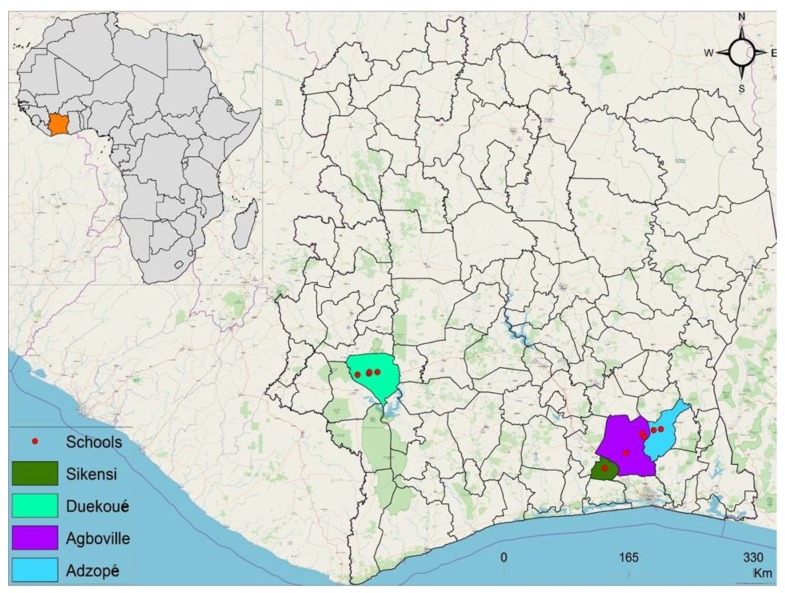
Collection of maps displaying the study area in Côte d’Ivoire, West Africa: Adzopé, Agboville, and Sikensi in the southern setting; Duekoué in the western setting of Côte d’Ivoire. Primary schools (red dots) were selected in each health district on the basis of close proximity to open freshwater bodies (distance: < 10 km).

The study was carried out in two settings of Côte d’Ivoire, including four health districts: (i) Agboville (geographical coordinates: 5° 55′ 41″ N latitude, 4° 13′ 01″ W longitude); (ii) Adzopé (6° 06′ 25″ N, 3° 51′ 36″ W); and (iii) Sikensi (5° 40′ 34″ N, 4° 34′ 33″ W), all located in the southern part of Côte d‘Ivoire; and (iv) Duekoué (6° 44′ 00″ N, 7° 21′ 00″ W) in the western part (Figure 1). We included one health district from the western part of Côte d’Ivoire in order to contrast with the three health districts in the South, thus enriching ecological features (the western part of Côte d’Ivoire is hilly as opposed to mainly flat terrain in the South) and biological characteristics (setting-specific parasite-intermediate host systems). In view of limited financial and human resources, we were unable to include the same amount of health districts in the western compared to the southern setting of Côte d’Ivoire.

Subsistence farming is the main economic activity in all study settings, which are well known for their endemicity of *S. haematobium* [16] and *S. mansoni* [12,17]. In each of the health districts, there are rivers which act as main transmission sites for schistosomiasis [18]. Of note, Duekoué is additionally appreciated as tourist destination. The Guemon River is the predominant river running right through the city. Sikensi has several rivers that discharge in the Agnéby River. Water flowing through Agboville also discharges in the Agnéby River [19]. Adzopé shares rivers from different catchment areas [18].

The study population consisted of schoolchildren aged 5–14 years. Schools were chosen on the basis of their proximity to a stream, river, lake, or backwater used by children at a distance of less than 10 km.

### 2.2. Design and Sample Size

A cross-sectional study was carried out in the two study settings from January to April 2018. The sample size (*n*) was adjusted to 1187 children based on the following formula: *n =* (*Z*^2^ × *p* (1 − *p*) × *C*) / *i*^2^, where *Z =* 1.96, *p =* 40% is the prevalence expected based on a previous study [9], *i* is the precision or margin of the error (5%), and *C* is the correction coefficient (*C =* 2).

### 2.3. Data and Sample Collection

Only schoolchildren aged 5–14 years who had lived in the study area for at least one year prior to the survey were included. The number of children per school was proportionally allocated according to population size in each school. Children were randomly selected using readily available school lists and identified by unique codes. A questionnaire was administered to collect data about each child’s habits and behaviors, such as swimming/bathing in open freshwater bodies, washing clothes in rivers, and fishing. In total, 1187 urine and 1089 stool samples were collected from children in plastic containers and transferred to nearest health centres for parasitological examination. Urine samples were collected between 10 a.m. and 12 a.m. [20].

### 2.4. Parasitological Examination

A urine filtration method was employed to identify *S. haematobium* eggs [21]. In brief, 10 mL of urine was vigorously shaken and filtered through a Nytrel filter with a 40 µm mesh size and examined microscopically for the presence of *S. haematobium* eggs that were counted by experienced laboratory technicians. Stool samples were subjected to the Kato-Katz technique [22]. Two thick smears from each stool sample were microscopically examined to identify and quantify eggs of *S. mansoni* and soil-transmitted helminths.

After examination, all schoolchildren were treated with a single 40 mg/kg oral dose of praziquantel (600 mg; Biltricide, Bayer, Leverkusen, Germany) through the "Programme National de Lutte contre les Maladies Tropicales Negligées à Chimioprophylaxie Préventive" (PNLMTN-CP) of Côte d’Ivoire. Children with soil-transmitted helminths were treated with albendazole (400 mg).

### 2.5. Statistical Analysis

Statistical analyses were performed with STATA version 15.0 (Stata Corporation; College Station, TX, USA). Univariate analysis (χ^2^ and Fisher’s exact test, as appropriate) was used for comparison between groups. Children were stratified into three age groups (5–8, 9–11, and 12–14 years). Parasitic infections were defined as positive for *S. haematobium* or *S. mansoni* when at least one egg was identified in a urine or stool sample, respectively. Associations between parasitic infections and sociodemographic, socioeconomic, or environmental factors were assessed by mixed multivariable logistic regression models with random intercepts for schools and for classes nested within schools. The study area was used as a fixed factor. The risk factors investigated were occupation and educational attainment of parents/legal guardians, and swimming, fishing, and playing in freshwater by children. Associations and differences with a *p*-value below 0.05 were considered statistically significant.

### 2.6. Ethical Consideration

Ethical clearance was obtained from the Ministère de la Santé et de l’Hygiène Publique de Côte d’Ivoire (reference no. 003–18/MSHP/CNER-kp). School authorities, teachers, parents/guardians, and participants were informed about the objectives, procedures, and potential risks and benefits of the study. Written informed consent was obtained from children’s parents or legal guardians. Oral assent was obtained from children.

## 3. Results

### 3.1. Sociodemographic Characteristics of the Population

A total of 1187 schoolchildren were included in the study. There were considerably more boys than girls (61.2% vs. 38.8%) and the highest proportion of children was included in Agboville. The mean age was 9.9 years (standard deviation (SD) = 2.4 years) with a median age of 10 years. Children aged 9–11 years were the most common age class in Adzopé (43.8%), Duekoué (40.3%), and Sikensi (54.1%) but the least common age class in Agboville (31.6%). Table 1 shows the sociodemographic characteristics of the study population, stratified by setting.

### 3.2. Urine and Stool Examination

#### 3.2.1. Infection with *S. haematobium*

All 1187 schoolchildren included in the study provided a single urine sample (100%). *S. haematobium* eggs were found in 166 of the children, owing to an overall prevalence of 14.0% (95% confidence interval (CI): 12.1%–16.1%). The prevalence of *S. haematobium* was considerably higher in the three school locations of the southern compared with the western Côte d’Ivoire (16.1% vs. 9.4%). The highest prevalence was found in Sikensi (35.6%). Boys and girls showed similar *S. haematobium* prevalence (14.2% vs. 13.7%; *p =* 0.781). No statistically significant difference was observed in the prevalence between age groups (*p* = 0.337).

Two cases of co-infection with *S. haematobium* and *S. mansoni* were found in Agboville. In two children from Duekoué, eggs identified in urine samples were morphologically determined as *S. mansoni* (Table 2).

#### 3.2.2. Infection with *S. mansoni*

Stool samples were obtained from 1089 children (91.7%). The overall prevalence of *S. mansoni* infection was 6.1% (95% CI: 4.8%–7.6%). *S. mansoni* was most commonly found in Agboville (11.2%), while no infections were found in Sikensi. Age and sex were not associated with *S. mansoni* infection (*p >* 0.05) (Table 2). The arithmetic mean of *S. mansoni* eggs per gram of stool (EPG), including standard error (SE) from positive samples, was 91.1 EPG (SE: 11.2 EPG; 95% CI: 68.7–113.4 EPG) with a minimum and maximum of 20 and 400 EPG, respectively. The geometric mean of *S. mansoni* eggs from positive stool samples was 4.1 (SD: 0.9).

#### 3.2.3. Other Helminths and Co-Infection

Three species of soil-transmitted helminths were identified in stool samples at very low rates: *Trichuris trichiura* (2.3%), *Ascaris lumbricoides* (1.7%), and hookworm (0.2%). In two school locations, children with concurrent *Schistosoma* and soil-transmitted helminth infections were identified; in Sikensi (*S. haematobium*-*T. trichiura* and *S. haematobium*-hookworm) and in Agboville (*S. mansoni*-*A. lumbricoides*, *S. mansoni*-*T. trichiura,* and triple species infection with *S. mansoni*, *A. lumbricoides,* and *T. trichiura*).

#### 3.2.4. Multivariate Logistic Regression Models

Table 3 shows the association between *Schistosoma* infection and sociodemographic factors, socioeconomic status, and environmental factors. The key risk factors for *S. haematobium* were swimming (adjusted odds ratio (AOR): 127.0; 95% CI: 25.0–634.0) and playing in water (AOR: 74.0; 95% CI: 3.8–144.3). For *S. mansoni*, children who lacked tap water at home (AOR: 2.7; 95% CI: 1.2–5.8) and who washed their clothes in open freshwater bodies (AOR: 5.3; 95% CI: 2.3–12.1) were the most infected.

The educational status as illiterate, of fathers (crude odds radio (COR): 3.3; 95% CI: 1.0–10.9) and mothers (COR: 11.5; 95% CI: 1.6–84.0) were also significantly associated with *S. mansoni* infection.

## 4. Discussion

This study was designed to determine the prevalence of the two known human *Schistosoma* species and to identify risk factors associated with infection among 5–14 year-old schoolchildren in southern and western parts of Côte d’Ivoire. We employed widely used diagnostic methods; namely a filtration method for detection and quantification of *S. haematobium* eggs in urine samples and the Kato-Katz technique for detection and quantification of *S. mansoni* (and soil-transmitted helminth) eggs in fecal samples. We found an overall prevalence of 14.0% for *S. haematobium* and 6.1% for *S. mansoni*, which classify our study settings as moderate and low endemic areas, respectively, for urogenital schistosomiasis and intestinal schistosomiasis, according to WHO guidelines [23]. The arithmetic mean of *S. mansoni* egg counts (91.9 EPG) recorded was low and would be classified as a light infection.

As expected, the current study reports a low prevalence of *S. haematobium* in Duekoué in the western part of Côte d’Ivoire, corroborating results from previous studies [11,17]. The highest prevalence of *S. mansoni* was found in Agboville, in one of the three school locations included in the southern part of Côte d’Ivoire. Similar studies showed the prevalence to be lower for *S. haematobium* (0.9%–4.4%), and higher for *S. mansoni* (17.5%–61.3%) in western Côte d’Ivoire [24]. Another study reported a high prevalence of *S. mansoni* (58.7%–68.4%) and low prevalence for *S. haematobium* (10.9%–18.4%) in southern Côte d’Ivoire [9]. The difference between the prevalence of the two schistosome species could be explained by the variation in ecological factors that influence the transmission dynamics. The low prevalence rate of *S. haematobium* and *S. mansoni* infections reported in our study is most likely the result of preventive chemotherapy campaigns pursued on an annual basis since several years by the PNLMTN-CP in Côte d’Ivoire[25].

We found similar prevalence rates for boys and girls, corroborating results from previous studies in Côte d’Ivoire [9]. However, it must be noted that the number of boys in our final study sample was considerably higher than that of girls (727 vs. 460), which was particularly pronounced in Sikensi in the southern part (142 vs. 63) and Duekoué in the western part of Côte d’Ivoire (240 vs. 132). This observation is in line with a large epidemiological study conducted in the late 1990s in the Man region of western Côte d’Ivoire. Among 12,227 children interviewed from 121 schools, there were 7489 boys and 4738 girls, pointing to a gender-bias in terms of school enrolment that tended to increase with age of children [26]. Interestingly, prior and current observations from Côte d’Ivoire are in contrast to a study from Senegal, where boys showed a higher prevalence of *Schistosoma* infection [27]. Results from other studies showed that when children are in contact with freshwater bodies for longer periods of time, they are more likely to be infected [9,28]. The risk of disease occurrence can increase because children are more often involved in recreational activities; hence, they are exposed to unprotected open surface freshwater for longer periods [29].

*S. mansoni* eggs, unmistakendly characterised by a lateral spine, were identified in two urine samples obtained from schoolchildren in Duekoué. The appearance of *S. mansoni* eggs in urine is unusual and has not been studied extensively [30]. In our investigation, *Schistosoma* species were determined by widely used methods based on egg morphology and light microscopy. Future studies should employ concurrent molecular approaches to improve diagnostic sensitivity.

In the current study, no differences in the prevalence of *Schistosoma* infection were observed among the three investigated age groups, which is in line with several other studies [20,31,32]. However, there are also studies reporting an increase in the prevalence with age of children [33,34]. Indeed, children aged 10–14 years can become more vulnerable for schistosomiasis during recreational activities, i.e., swimming and playing in water, or while fetching water for household use, or agriculture activities [35,36]. Most of the children in our study who did not have tap water at home were infected by *S. mansoni*, contrary to results reported from South Africa [34].

Socioeconomic factors were significantly associated with the occurrence of schistosomiasis. In particular, a significant relationship between illiteracy of the parents/guardians and *S. mansoni* infection was reported. Similar results were found in a previous study in Nigeria, which showed that better educated parents can understand the preventive campaigns more deeply and thus better explain them to their children [35].

The association between the occurrence of schistosomiasis and contact with freshwater bodies is well documented. In our study, swimming and fishing in freshwater by schoolchildren was strongly associated with infection with *S. haematobium*. This might be explained by the fact that, while swimming, infected children emit urine with *S. haematobium* eggs. The eggs hatch, infecting snails which produce furcocercariae that penetrate the skin of children exposed to the contaminated water. This corroborates the findings of other studies that reported high infection rates among children who swim in rivers [25,37]. Other researchers did not report a significant correlation between swimming and occurrence of schistosomiasis [20,38]. Our finding that washing clothes in water and lack of tap water at home were associated with the occurrence of *S. mansoni* infection is in line with results from other studies [9,34].

The low prevalence of any of the three common soil-transmitted helminths in our study confirms observations from previous surveys [39,40]. This observation is likely attributed to large-scale preventive chemotherapy campaigns by the PNLMTN-CP, coupled with systematic sensitization and deworming carried out by non-governmental organizations (NGOs) and improvements in sanitation in face of social and economic development.

The current study has several limitations, and hence, the findings should be interpreted with care. First, stool and urine samples were collected only on a single day, though duplicate Kato-Katz thick smears were examined from each stool sample to enhance diagnostic sensitivity. It is conceivable that a number of infections, particularly those of light intensity were missed, and hence, the overall prevalence of both *S. haematobium* and *S. mansoni* might be somewhat higher than reported here [41]. Second, data about sociodemographic, socioeconomic, and environmental factors were collected through a pretested questionnaire administered to children. There might be some kind of reporting bias. Third, no specific information was collected on water, sanitation, and hygiene behavior, although these are known risk factors for schistosomiasis [42,43].

## 5. Conclusions

Our study confirms that schistosomiasis remains prevalent in the southern and western parts of Côte d’Ivoire, although the overall prevalence in school-aged children was much lower than reported a few years earlier. Swimming, washing clothes, playing in unprotected open freshwater bodies, and low educational attainment of parents/guardians were identified as key risk factors of schistosomiasis in schoolchildren. Hence, preventive chemotherapy using praziquantel—which is the current mainstay of the national schistosomiasis control program—should be combined with targeted information and education campaigns to change children’s behaviors, with the goal of reducing the frequency of contact of children with open freshwater bodies.

## Figures and Tables

**Table 1:** Sociodemographic characteristics of the study population subjected to schistosomiasis diagnosis in different settings of Côte d’Ivoire in early 2018.

Variable	Western Setting	Southern Setting			Total (N = 1187)
	Duekoué (*n* = 372)	Adzopé (*n* = 208)	Agboville (*n* = 402)	Sikensi (*n* = 205)	
**Sex**					
Girl (%)	132 (35.5)	83 (39.9)	182 (45.3)	63 (30.7)	460 (38.8)
Boy (%)	240 (64.5)	125 (60.1)	220 (54.7)	142 (69.3)	727 (61.2)
**Age (years)**					
5–8 (%)	109 (29.3)	55 (26.4)	135 (33.6)	58 (28.3)	357 (30.1)
9–11 (%)	150 (40.3)	91 (43.8)	127 (31.6)	111 (54.1)	479 (40.5)
12–14 (%)	113 (30.4)	62 (29.8)	140 (34.8)	36 (17.6)	351 (29.4)

*n*: number of children included in each study site; N: number of children included overall; %: percentage in each category.

**Table 2:** Prevalence rate of *Schistosoma haematobium* and *Schistosoma mansoni* infection, stratified by study settings, sex, and age group among schoolchildren from Côte d’Ivoire in early 2018.

Characteristic	*S. haematobium*		*S. mansoni*	
	Total	Positive *n* (%)	Total	Positive *n* (%)
**Western setting**				
Duekoué	372	35 (9.4)^2^	274	20 (7.3)
**Southern setting**				
Adzopé	208	22 (10.6)	208	1 (0.9)
Agboville	402	36 (9.0)^1^	402	45 (11.2)^1^
Sikensi	205	73 (35.6)	205	0
*p-*value		< 0.001		< 0.001
**Sex**				
Boy	727	103 (14.2)	408	27 (6.6)
Girl	460	63 (13.7)	681	39 (5.7)
*p-*value		0.781		0.551
**Age (years)**				
5–8	357	60 (16.8)	307	15 (4.9)
9–11	479	65 (13.6)	443	24 (4.4)
12–14	351	41 (11.7)	339	27 (8.0)
*p-*value		0.337		0.199

^1^: Two children were co-infected with *S. haematobium* and *S. mansoni.* ^2^: Two of these 35 positive cases were identified microscopically as *S. mansoni* eggs. *n*: number of positive children after microscopic examination

**Table 3:** Multivariate logistic regression model analysis of variables associated with *S. haematobium* and *S. mansoni* infection among schoolchildren non-adjusted and adjusted for sociodemographic factors, socioeconomic status, and environmental factors.

Characteristics	*S. haematobium*				*S. mansoni*			
	Total	Positive	Crude OR (95% CI)	Adjusted OR (95% CI)	Total	Positive	Crude OR (95% CI)	Adjusted OR (95% CI)
**Sociodemographic factors**								
*School locations*								
Agboville	402	36	1.00	1.00	402	45	0.04 (0.01–0.28)	1.88 (0.02–2.20)
Adzopé	208	22	1.20 (0.69–2.10)	1.72 (0.25–11.61)	208	1	1.00	1.00
Duekoué	372	35	1.06 (0.65–1.72)	0.67 (0.10–4.37)	274	20	0.62 (0.36–1.08)	1.66 (0.54–5.10)
Sikensi	205	73	5.62 (3.60–8.78)*	4.43 (0.10–198.43)	205	0	–	–
***Sex***								
Girl	460	63	1.00	1.00	408	27	1.00	1.00
Boy	727	103	1.04 (0.74–1.46)	0.92 (0.32–2.68)	681	39	0.86 (0.52–1.42)	0.98 (0.52–1.82)
*Age (years)*								
5–8	357	60	1.00	1.00	307	15	1.00	1.00
9–11	479	65	0.78 (0.53–1.14)	0.15 (0.02–0.99)	443	24	1.11 (0.58–2.16)	0.37 (0.10–1.35)
12–14	351	41	0.65 (0.42–1.00)	0.28 (0.03–2.80)	339	27	1.68 (0.88–3.23)	0.23 (0.05–1.09)
**Socioeconomic factors**								
*Father’s education*								
Illiterate	710	104	1.30 (0.69–2.46)	1.20 (0.05–28.41)	620	57	3.34 (1.03–10.88)*	–
Primary school	101	12	1.02 (0.44–2.40)	1.15 (0.01–25.70)	101	0	–	–
Secondary school	104	12	0.99 (0.42–2.32)	0.45 (0.01–6.46)	104	1	0.32 (0.03–3.13)	–
Expert level	103	12	1.00	1.00	102	3	1.00	–
*Mother’s education*								
Illiterate	856	139	3.39 (0.46–26.35)	3.18 (0.03–26.50)	767	58	11.54 (1.58–83.96)*	–
Primary school	142	17	2.45 (0.31–19.53)	3.98 (0.04–38.00)	142	1	0.42 (0.10–4.54)	–
Secondary school	51	5	1.96 (0.21–17.93)	0.34 (0.04–28.20)	53	0	–	–
Expert level	19	1	1.00	1.00	18	1	1.00	–
*Father’s occupation*								
Farmer	735	123	1.00	1.00	694	48	1.00	1.00
Fisherman	152	20	0.75 (0.45–1.25)	2.26 (0.57–9.00)	105	6	0.82 (0.34–1.96)	0.72 (0.17–3.07)
Official	269	21	0.42 (0.26–0.68)	0.23 (0.01–3.56)	265	10	0.53 (0.26–1.06)	1.08 (0.33–3.51)
*Mother’s occupation*								
Farmer	617	115	1.00	1.00	551	42	1.00	1.00
Householder	520	49	0.45 (0.32–0.65)	1.13 (0.32–4.05)	495	23	0.59 (0.35–1.00)	1.13 (0.42–3.01)
Official	22	1	0.21 (0.03–1.56)	0.63 (0.10–9.90)	21	0	–	–
**Environmental factors**								
*Using tap water*								
Yes	539	74	1.00	1.00	447	17	1.00	1.00
No	618	91	1.08 (0.78–1.51)	0.55 (0.14–2.17)	616	49	2.19 (1.24–3.85)	2.65 (1.22–5.79)*
*Swimming*								
No	922	1	1.00	1.00	879	63	1.00	1.00
Yes	265	165	152 (21–1097)	127 (25–634)*	210	3	0.18 (0.06–0.60)	0.35 (0.09–1.40)
*Washing clothes*								
No	118	4	1.00	1.00	917	38	1.00	1.00
Yes	1069	162	0.20 (0.07–0.54)	0.70 (0.10–82.0)	118	28	7.64 (4.48–13.02)	5.26 (2.28–12.10)*
*Fishing*								
No	386	40	1.00	1.00	338	9	1.00	1.00
Yes	801	126	1.61 (1.11–2.36)	74.0 (3.8–144.3)*	751	57	3.00 (1.47–6.14)	1.88 (0.34–10.33)
*Playing*								
No	493	55	1.00	1.00	432	12	1.00	1.00
Yes	694	111	1.52 (1.07–2.14)	1.98 (0.09–45.32)	657	54	3.13 (1.66–5.93)	0.61 (0.11–3.25)

* *p*-value < 0.05, *p*-value obtained from a mixed logistic regression model with fixed effects for the prevalence of *S. haematobium* or *S. mansoni* and each variable in the table.

## References

[1] Hotez P.J., Alvarado M., Basáñez M.-G., Bolliger I., Bourne R., Boussinesq M., Brooker S.J., Brown A.S., Buckle G., Budke C.M. (2014). The Global Burden of Disease Study 2010: Interpretation and implications for the neglected tropical diseases. PLoS Negl. Trop. Dis..

[2] GBD 2017 DALYs and Hale Collaborators (2018). Global, regional, and national disability-adjusted life-years (DALYs) for 359 diseases and injuries and healthy life expectancy (HALE) for 195 countries and territories, 1990-2017: A systematic analysis for the Global Burden of Disease study 2017. Lancet.

[3] McManus D.P., Dunne D.W., Sacko M., Utzinger J., Vennervald B.J., Zhou X.-N. (2018). Schistosomiasis. Nat. Rev. Dis. Primer.

[4] Colley D.G., Bustinduy A.L., Secor W.E., King C.H. (2014). Human schistosomiasis. Lancet.

[5] Aagaard-Hansen J., Mwanga J.R., Bruun B. (2009). Social science perspectives on schistosomiasis control in Africa: Past trends and future directions. Parasitology.

[6] Utzinger J., N’Goran E.K., Caffrey C.R., Keiser J. (2011). From innovation to application: Social-ecological context, diagnostics, drugs and integrated control of schistosomiasis. Acta Trop..

[7] Steinmann P., Keiser J., Bos R., Tanner M., Utzinger J. (2006). Schistosomiasis and water resources development: Systematic review, meta-analysis, and estimates of people at risk. Lancet Infect. Dis..

[8] McCreesh N., Nikulin G., Booth M. (2015). Predicting the effects of climate change on *Schistosoma mansoni* transmission in eastern Africa. Parasit. Vectors.

[9] Coulibaly J.T., N’Gbesso Y.K., N’Guessan N.A., Winkler M.S., Utzinger J., N’Goran E.K. (2013). Epidemiology of schistosomiasis in two high-risk communities of south Côte d’Ivoire with particular emphasis on pre-school–aged children. Am. J. Trop. Med. Hyg..

[10] Tian-Bi Y.-N.T., Webster B., Konan C.K., Allan F., Diakité N.R., Ouattara M., Salia D., Koné A., Kakou A.K., Rabone M. (2019). Molecular characterization and distribution of *Schistosoma* cercariae collected from naturally infected bulinid snails in northern and central Côte d’Ivoire. Parasit. Vectors.

[11] Chammartin F., Houngbedji C.A., Hürlimann E., Yapi R.B., Silué K.D., Soro G., Kouamé F.N., N’Goran E.K., Utzinger J., Raso G. (2014). Bayesian risk mapping and model-based estimation of *Schistosoma haematobium*-*Schistosoma mansoni* co-distribution in Côte d’Ivoire. PLoS Negl. Trop. Dis..

[12] Utzinger J., N’Goran E.K., Tanner M., Lengeler C. (2000). Simple anamnestic questions and recalled water-contact patterns for self-diagnosis of *Schistosoma mansoni* infection among schoolchildren in western Côte d’Ivoire. Am. J. Trop. Med. Hyg..

[13] Assaré R.K., Lai Y.-S., Yapi A., Tian-Bi Y.-N.T., Ouattara M., Yao P.K., Knopp S., Vounatsou P., Utzinger J., N’Goran E.K. (2015). The spatial distribution of *Schistosoma mansoni* infection in four regions of western Côte d’Ivoire. Geospat. Health.

[14] Soumahoro M., Bosson-Vanga A., Coulibaly K., Abbes S., Angora E., Kouadio K., N’Douba A.K., Sissoko D., Dosso M. (2014). Investigation d’un foyer épidémique de bilharziose urinaire dans l’école primaire du village de Guébo 2, Abidjan, Côte d’Ivoire. Bull. Soc. Pathol. Exot..

[15] M’Bra R.K., Kon_ B., Yapi Y.G., Silué K.D., Sy I., Vienneau D., Soro N., Cissé G., Utzinger J. (2018). Risk factors for schistosomiasis in an urban area in northern Côte d’Ivoire. Infect. Dis. Poverty.

[16] N’Guessan N., Acka C.A., Utzinger J., N’Goran E.K. (2007). Identification des régions à haut risque de schistosomoses en Côte d’lvoire. Bull. Soc. Pathol. Exot..

[17] Raso G., Matthys B., N’Goran E.K., Tanner M., Vounatsou P., Utzinger J. (2005). Spatial risk prediction and mapping of *Schistosoma mansoni* infections among schoolchildren living in western Côte d’Ivoire. Parasitology.

[18] Nwaorgu O.C., Okeibunor J., Madu E., Amazigo U., Onyegegbu N., Evans D. (1998). A school-based schistosomiasis and intestinal helminthiasis control programme in Nigeria: Acceptability to community members. Trop. Med. Int. Health.

[19] Diakité N.R., N’Zi K.G., Ouattara M., Coulibaly J.T., Saric J., Yao P.K., Hattendorf J., Utzinger J., N’Goran E.K. (2018). Association of riverine prawns and intermediate host snails and correlation with human schistosomiasis in two river systems in south-eastern Côte d’Ivoire. Parasitology.

[20] Geleta S., Alemu A., Getie S., Mekonnen Z., Erko B. (2015). Prevalence of urinary schistosomiasis and associated risk factors among Abobo primary school children in Gambella Regional State, southwestern Ethiopia: A cross sectional study. Parasit. Vectors.

[21] Mott K.E., Baltes R., Bambagha J., Baldassini B. (1982). Field studies of a reusable polyamide filter for detection of *Schistosoma haematobium* eggs by urine filtration. Tropenmed. Parasitol..

[22] Katz N., Chaves A., Pellegrino J. (1972). A simple device for quantitative stool thick-smear technique in schistosomiasis *mansoni*. Rev. Inst. Med. Trop. Sao Paulo.

[23] (2006). WHO Preventive Chemotherapy in Human Helminthiasis: Coordinated Use of Anthelminthic Drugs in Control Interventions: A Manual for Health Professionals and Programme Managers.

[24] Yapi Y.G., Briët O.J.T., Diabate S., Vounatsou P., Akodo E., Tanner M., Teuscher T. (2005). Rice irrigation and schistosomiasis in savannah and forest areas of Côte d’Ivoire. Acta Trop..

[25] Tian-Bi Y.-N.T., Ouattara M., Knopp S., Coulibaly J.T., Hürlimann E., Webster B., Allan F., Rollinson D., Meïté A., Diakité N.R. (2018). Interrupting seasonal transmission of *Schistosoma haematobium* and control of soil-transmitted helminthiasis in northern and central Côte d’Ivoire: A SCORE study protocol. BMC Public Health.

[26] Utzinger J., N’Goran E.K., Ossey Y.A., Booth M., Traoré M., Lohourignon K.L., Allangba A., Ahiba L.A., Tanner M., Lengeler C. (2000). Rapid screening for *Schistosoma mansoni* in western Côte d’Ivoire using a simple school questionnaire. Bull. World Health Organ..

[27] Sow S., de Vlas S.J., Stelma F., Vereecken K., Gryseels B., Polman K. (2011). The contribution of water contact behavior to the high *Schistosoma mansoni* infection rates observed in the Senegal River Basin. BMC Infect. Dis..

[28] Diakité N.R., Winkler M.S., Coulibaly J.T., Guindo-Coulibaly N., Utzinger J., N’Goran E.K. (2017). Dynamics of freshwater snails and *Schistosoma* infection prevalence in schoolchildren during the construction and operation of a multipurpose dam in central Côte d’Ivoire. Infect. Dis. Poverty.

[29] Kazibwe F., Makanga B., Rubaire-Akiiki C., Ouma J., Kariuki C., Kabatereine N.B., Vennervald B.J., Rollinson D., Stothard J.R. (2010). Transmission studies of intestinal schistosomiasis in Lake Albert, Uganda and experimental compatibility of local *Biomphalaria* spp.. Parasitol. Int..

[30] Ratard R., Ndamkou C., Kouemeni L., Ekani Bessala M. (1991). *Schistosoma mansoni* eggs in urine. J. Trop. Med. Hyg..

[31] Abou-Zeid A.H., Abkar T.A., Mohamed R.O. (2013). Schistosomiasis infection among primary school students in a war zone, Southern Kordofan State, Sudan: A cross-sectional study. BMC Public Health.

[32] Negussu N., Wali M., Ejigu M., Debebe F., Aden S., Abdi R., Mohamed Y., Deribew A., Deribe K. (2013). Prevalence and distribution of schistosomiasis in Afder and Gode zone of Somali region, Ethiopia. J. Glob. Infect. Dis..

[33] Ivoke N., Ivoke O.N., Nwani C.D., Ekeh F.N., Asogwa C.N., Atama C.I., Eyo J.E. (2014). Prevalence and transmission dynamics of *Schistosoma haematobium* infection in a rural community of southwestern Ebonyi State, Nigeria. Trop. Biomed..

[34] Kabuyaya M., Chimbari M.J., Mukaratirwa S. (2018). Infection status and risk factors associated with urinary schistosomiasis among school-going children in the Ndumo area of Mkhanyakude district in KwaZulu-Natal, South Africa two years post-treatment. Int. J. Infect. Dis..

[35] Ugbomoiko U.S., Ofoezie I.E., Okoye I.C., Heukelbach J. (2010). Factors associated with urinary schistosomiasis in two peri-urban communities in south-western Nigeria. Ann. Trop. Med. Parasitol..

[36] Sady H., Al-Mekhlafi H.M., Mahdy M.A.K., Lim Y.A.L., Mahmud R., Surin J. (2013). Prevalence and associated factors of schistosomiasis among children in Yemen: Implications for an effective control programme. PLoS Negl. Trop. Dis..

[37] Coulibaly G., Ouattara M., Dongo K., Hürlimann E., Bassa F.K., Koné N., Essé C., Yapi R.B., Bonfoh B., Utzinger J. (2018). Epidemiology of intestinal parasite infections in three departments of south-central Côte d’Ivoire before the implementation of a cluster-randomised trial. Parasite Epidemiol. Control.

[38] Ayele B., Erko B., Legesse M., Hailu A., Medhin G. (2008). Evaluation of circulating cathodic antigen (CCA) strip for diagnosis of urinary schistosomiasis in Hassoba school children, Afar, Ethiopia. Parasite.

[39] Abossie A., Seid M. (2014). Assessment of the prevalence of intestinal parasitosis and associated risk factors among primary school children in Chencha town, Southern Ethiopia. BMC Public Health.

[40] Nundu Sabiti S., Aloni M.-N., Linsuke S.-W.-L., Ekila M.-B., Situakibanza H.-T., Polman K., Lutumba P.-T. (2014). Prevalence of geohelminth infections in children living in Kinshasa. Arch. Pediatr..

[41] Bärenbold O., Raso G., Coulibaly J.T., N’Goran E.K., Utzinger J., Vounatsou P. (2017). Estimating sensitivity of the Kato-Katz technique for the diagnosis of *Schistosoma mansoni* and hookworm in relation to infection intensity. PLoS Negl. Trop. Dis..

[42] Hilali A.H., Madsen H., Daffalla A.A., Wassila M., Christensen N.O. (1995). Infection and transmission pattern of *Schistosoma mansoni* in the Managil irrigation scheme, Sudan. Ann. Trop. Med. Parasitol..

[43] Grimes J.E.T., Croll D., Harrison W.E., Utzinger J., Freeman M.C., Templeton M.R. (2014). The relationship between water, sanitation and schistosomiasis: A systematic review and meta-analysis. PLoS Negl. Trop. Dis..

